# A framework for evaluating the impact of the United Nations fellowship programmes

**DOI:** 10.1186/1478-4491-8-7

**Published:** 2010-03-30

**Authors:** Arie Rotem, Michael A Zinovieff, Alexandre Goubarev

**Affiliations:** 1 School of Public Health and Community Medicine, UNSW, Sydney, Australia; 2 Consultant in Human Resources, Geneva, Switzerland; 3 Department of Human Resources for Health, WHO, Geneva

## Abstract

The United Nations (UN) System's agencies have been criticized for not adequately assessing the impact of their training and fellowship programmes. Critics point out that beyond documentation of the number of fellows that underwent training, and their immediate reaction to the experience, it is necessary to ascertain that fellows are using what they have learned, and most importantly that their institution and country are benefiting from the significant investments made in the fellowship programmes.

This paper presents an evaluation framework that was adopted by the 17th Meeting of the UN System Senior Fellowship Officers convened in London in 2008 in response to this challenge. It is arranged in three sections. First, the assumptions and constraints concerning impact evaluation of training are presented. Second, a framework for evaluating the impact of training in the context of UN System programmes is proposed. Third, necessary conditions and supportive measures to enable implementation of the impact evaluation framework are identified.

The critical message emerging from this review is the importance of constructing a 'performance story' based on key milestones associated with the design and implementation of fellowship programmes as a way of assessing the contribution of different components of the fellowship programmes to institutional outcomes.

## Background

The United Nations (UN) System's agencies have been criticized for not adequately assessing the impact of their training and fellowship programmes [1]. Critics point out that beyond documentation of the number of fellows that underwent training, and their immediate reaction to the experience, it is necessary to ascertain that fellows are using what they have learned, and most importantly that their institution and country are benefiting from the significant investments made in the fellowship programmes.

In response to these concerns, the 16^th ^Meeting of the UN System Senior Fellowship Officers (Paris, November 2006) mandated the design of a generic evaluation framework that defines the scope, dimensions and core indicators for evaluating the impact of UN Fellowship programmes [2].

This paper presents an evaluation framework that was adopted by the 17th Meeting of the UN System Senior Fellowship Officers (London, November 2008). It is arranged in three major sections. First, the assumptions and constraints concerning impact evaluation of training are presented. Second, a framework for evaluating the impact of training in the context of UN System programmes is proposed. Third, necessary conditions and supportive measures to enable implementation of the impact evaluation framework are identified.

## Modalities of fellowships

In its detailed and well received 1998 report on "Fellowships in the United Nations System" the Joint Inspection Unit (JIU) proposed the following definition;

"...a fellowship in the United Nations system is a specially tailored or selected training activity that provides a monetary grant to a qualified individual or group of qualified individuals for the purpose of fulfilling special learning objectives; such training may be of short or long duration and may take place in an appropriate training institution or in the field inside or outside the fellow's country; should be in response to nationally-approved human resources policies and plans and should aim at impact and relevance for all stakeholders involved [3].

It should be noted that the JIU definition has not been accepted by all UN agencies. Some agencies exclude study tours, while others actually include seminars and workshops. Moreover, the financial commitment of fellowships has not been accepted by all. Some bilateral and multilateral institutions outside the UN system put the emphasis on the extent to which any fellowships modality contributes to the achievement of clearly defined organizational objectives rather than on the definition of fellowships. Such a determination can only be ventured on the basis of evaluation of the effectiveness of fellowships in all its modalities. Noting that evaluation is the Achilles heel in most UN organizations, the JIU report acknowledge that effectiveness of fellowship in all its modalities is fundamentally linked to assessing its benefits to individuals and institutions.

## Evaluating fellowship programmes

Classification of different type of measures abound. In the context of training, none is more influential than Kirkpatrick [4,5], who proposed in the late fifties a framework for evaluating training using four levels of measurement:

a) Reaction - a measure of satisfaction (what the trainees/fellows thought and felt about the training);

b) Learning - a measure of learning (the resulting increase in knowledge or capability as reflected in end of course assessment);

c) Behaviour - a measure of behaviour change (extent of behaviour and capability improvement as reflected in on the job performance);

d) Results - a measure of results (the effects on the institutional environment resulting from the fellows' performance).

Kirkpatrick followers have suggested additional levels including, for example the introduction of a fifth level concerning estimation of the Return on Investment (ROI) [6]. Other useful additions before and after training include an assessment of the planning, design and implementation of the training programme and evaluation of the long term benefits to particular target groups and the social system at large.

Basing an impact evaluation framework on the strength of Kirkpatrick work mandates two important observations. First, it is necessary to note that most of Kirkpatrick's and his followers work were undertaken in the context of corporate training, whereby the trainees were also employees trained for well defined purposes. In these circumstances it has been possible to assess the training outcomes in relation to institutional key results areas and to estimate the returns on training investments. UN System fellows, on the other hand, return to different organisations and the impact of their learning on their home institutions is infinitely more difficult to assess due to limited control over their deployment and support once the fellowship is completed.

The second observation is even more pertinent. Although Kirkpatrick's 4 step approach has been widely discussed in the literature, it is evident that most organisations have not evaluated all four levels. Training interventions have been typically evaluated at the reaction and learning levels. Only a few studies have paid attention to behavioural outcomes, and very few assessed the benefit to organisations. The reliance on fellows' reaction and learning measures may reflect the difficulty and cost associated with measuring performance and organizational benefits and may underpin the limitation of current approaches [7].

## Recognising the constraints

Key constraints associated with the assessment of the impact of training include:

### Methodological constraints

(a) Methodological constraints associated with the attribution of any impact or change in the performance of individuals or systems to the participation in fellowship programmes. Reporting results and 'proving' attribution are two different things. Attribution involves drawing causal links and explanatory conclusions between observed changes and specific interventions [8]. If we wish to draw conclusions about the value of the programme and make decisions about its future direction we are expected to demonstrate that the programme contributed to the attainment of particular outcomes [9]. These links could be relatively easy to establish at a product or output level. It is much more difficult to attribute impact at higher levels (programme, agency, sectoral or national outcomes), or in complex systems. Determining whether an outcome was caused by a particular programme, partner programmes, other donor activities, or societal change, is difficult to substantiate.

### Conceptual constraints

(b) Conceptual constraints associated with the expectation that training on its own would have a sustainable impact on the system evaluated. Change in performance is commonly based on a multi prong intervention of which "people development" is only one of the elements. Conceptually there is no basis to expect that training alone would influence the performance of complex systems in the absence of contributing factors such as appropriate technology, resources, leadership, favourable internal and external conditions, conducive formal structure and most importantly supportive organizational culture.

### Programme fidelity

(c) The challenge of programme me fidelity. Experience with some UN System programmes have shown that fellowships are not always linked to well articulated objectives, that the selection process may be skewed, that the host institutions and programmes often lack understanding of the training needs, that fellows return to settings that fail to support and utilize them properly, and other such deficiencies. Furthermore, there is a great variability in the design and implementation of fellowship programmes in terms of duration, mode of training, recipient instruction resources and capacity and other variables. The fidelity of the fellowship programme as reflected in the presence of these critical determinants should arguably be demonstrated before major investments are made in the measurement of impact.

## Addressing common constraints

Whilst it is not possible to completely eliminate these constraints, it may be possible to mitigate their effect. The mapping of the entire pathway, from the initial identification of training needs and selection of fellows to the assessment of the support for and utilization of fellows in the home institution provides what Brinkerhoff and Gill [10] called an "impact map". The milestones signalled in this kind of map may help to verify the fidelity of the programme and at the same time help to identify the factors that affect progress towards outcomes.

In turn, the mapping of the fellowship programme pathway will improve our understanding of the added contribution of each component of the fellowship programme, helping us to attribute their contribution to the overall impact and improving our understanding of the role of training in the context of a broader capacity building effort. Most importantly, it should help us to verify that fellowship programmes have clear and well understood objectives in line with recipient institutions' priorities, that the programmes were well designed and funded, that the right people were selected and participated, that the training they received was consistent with their work settings requirements, that they gained the skill and knowledge they required, that they returned home and were posted in relevant positions, that they were given necessary support and opportunity, that they were able to apply their newly acquired competencies in practice, that their performance lead to improvement in programmes and institutional performance and ultimately that the programmes benefited particular customers or communities. In theory each step is a logical necessary condition for the success of subsequent steps. We could view these steps as a 'hierarchy of outcomes' we wish to achieve and hence need to monitor and evaluate.

## The contribution analysis approach

The notion of mapping the pathway towards higher level goals is consistent with Mayne's Contribution Analyses approach [11]. According to Kotvojs and Shrimpton [12], who have applied the contribution analysis in the context of an international development aid project, contribution analyses could address the challenge of attribution and verification of the logic of any programme (what we could reasonably expect). The approach is well suited to development programmes where data is likely to reflect 'progress toward results', rather than a definitive statement of final outcomes. As they point out "..., there is no expectation in Mayne's approach that causality can be firmly established, or that assessing a programme's contribution to outcomes should be conducted solely through quantitative methods. Mayne's [9] broader approach to Contribution Analysis seeks to achieve what Hendricks calls a 'plausible association', whereby a 'reasonable person, knowing what has occurred in the programme and that intended outcomes actually occurred, agrees that the programme contributed to those outcomes' (cited in [9]).

Thus, as Mayne [9] suggested, developing a results chain, and assessing alternative explanations for outcomes, enables us to produce a plausible 'performance story', and in turn, to estimate the degree to which results could be attributed to particular interventions. As Iverson [8] has noted, "contribution analysis accepts that in order to create a 'credible picture of attribution', complexity is recognised, multiple influences acknowledged and mixed methods used to 'gain (an) understanding of what programmes work, what parts of which programmes work, why they worked, and in what contexts"'. (Cited in [12])

## Systematic review of the pathway towards higher goals

Whilst most UN System agencies have monitored some of the key milestones related to the result chain of a fellowship programme, it appears that the link between these steps as a pathway leading to impact has not been explored systematically. The Logframe approach which is used by many agencies in project design and monitoring, points in the right direction. By linking higher level goals to specific objectives, activities and inputs, the framework enables clarity about the pathway. The inclusion of indicators for verification of progress and attainment of goals provides a blue print for monitoring and evaluation and the identification of barriers along the way.

The World Bank evaluation indicators [13] which were applied to the fellowship context by the WHO Senior Fellowship Officers make a good contribution in this regard. The WB framework provides a number of dimensions that are consistent with the notion of 'milestones' discussed above. For example, verifying that the selection of participants to a fellowship programme is based on priority needs addresses indicators related to 'Relevance'. Review of the design and implementation of the fellowship programme may address the criteria of 'Effectiveness', 'Efficiency' and 'Sustainability'. The validation of these criteria opens the way for further exploration concerning the longer term impact of fellowships on the performance of institutions and services.

The critical message emerging from these assumptions and assertions is the importance of constructing a 'performance story' based on key milestones associated with the design and implementation of fellowship programmes, as a way of assessing the contribution of different components of the fellowship programmes to institutional outcomes [14,15].

## A generic framework for evaluating the impact of fellowship programmes

The proposed framework for evaluating the impact of UN System fellowships is based on an attempt to analyse the contribution of different events and experiences to the attainment of particular results. The emerging performance story enables 'reasonable' observers to determine the plausibility that particular interventions led to certain results. The performance story describes the journey from the inception of a fellowship programme to the attainment of its immediate and long term goals. The important events and experiences along the way are identified as milestones (performance indicators) that are monitored in order to ascertain that the programme is moving in the right direction and ultimately it has reached its destination. (See Additional File 1 for a summary of sample indicators and methods of data collection that may be used to review the key milestones).

The verification that certain milestones have been reached strengthens our confidence in the contribution of particular outcomes towards the results (attribution) and reduces uncertainties associated with alternative explanations. In addition, the proposed analysis aims to explore the fidelity of the fellowship programme by ascertaining the fulfilment of necessary steps that lead us to expect that the fellowship intervention produce benefits to the recipient institutions.

The proposed evaluation framework focuses on both qualitative and quantitative evidence concerning the attainment of milestones implied in the extended Kirkpatrick classification of training evaluation measures. In addition to the four domains suggested by Kirkpatrick (reaction, learning, behaviour, results) we include the domains of planning, design and implementation and the long term impact ('mega-impact') of the fellowship programme.

The data collection methods are varied to provide a triangulation that may increase our confidence in the emerging findings. We stress the benefit of using existing information through secondary analysis of records and reports. Information routinely kept by institutions and fellowship authorities, is more economical to obtain and less likely to be biased. Accessing existing data, however, requires close collaboration with recipient institutions, fellowship authorities and other relevant stakeholders. Engagement of stakeholders in the elicitation of information and the interpretation of what it means is an essential component of this approach, as we rely on a deeper understanding of contextual issues and conditions which may affect the results. Furthermore, the involvement of stakeholders supports the emergence of ownership, an essential condition for capacity building and lasting improvement.

## Key milestones pathway for impact assessment of fellowships

**Figure 1:**
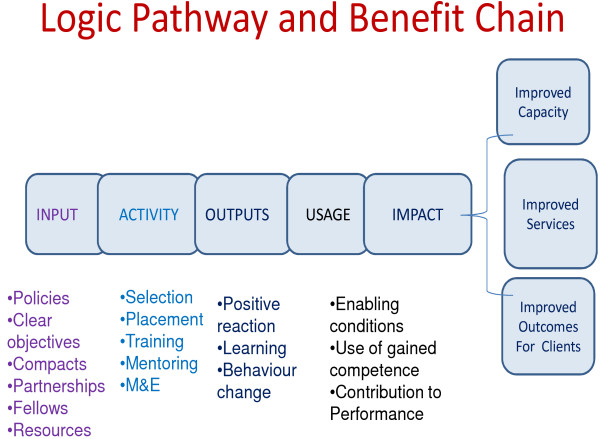
**Logic Pathway and Benefit Chain to be monitored to ascertain the benefits of the fellowship programme**.

Figure 1 presents the logic pathway and benefit chain that should be monitored to ascertain the benefits of the fellowship program.

Key indicators include:

• Clear objectives:

- aligned with national priorities and UN Agencies' mandates.

- based on training needs analysis.

- articulated in an achievable and cost effective education and training plan.

• Fair and transparent selection of fellows based on established selection criteria

• Relevant and appropriate placement of fellows using host institution with relevant expertise and adequate resources to provide an effective and efficient programme

• Successful and timely completion of fellows' programme:

- accomplished education and training objectives and certification of competence, where applicable (Evidence of learning gained through the fellowship).

- resulting in positive feedback from fellows and other stakeholders (found the learning experience suitable and beneficial and would recommend similar arrangements to their colleagues).

• Return home to relevant position with adequate support:

- percentage of fellows who are employed in relevant positions following various intervals of time.

- level and appropriateness of support provided to returning fellows (mentoring).

• Evidence of positive contribution to work:

- self and others' reports about enhanced capacity and contribution with concrete and verifiable examples (changes in behaviour or performance that could be reasonably attributed to the learning experience offered by the fellowship).

- continuing professional and personal development, and contribution to others' learning (dissemination).

- increasing productivity.

• Evidence of positive development in performance:

- examples of new programmes or innovative ways of working (including new technologies) that led to more effective performance.

- bridging operational gaps.

- strengthened professional networks.

• Improved performance leading to enhanced services and benefits provided to community:

- evidence concerning benefits to the target community.

- contribution to attainment of development goals including MDGs.

## Supportive conditions for effective implementation of impact evaluation of fellowships programmes

The third objective of this paper is to identify necessary conditions and supportive measures to enable implementation of the impact evaluation framework in the context of the UN System fellowships programmes. Our comments and recommendations here are made with reference to the application of contribution analysis presented in the previous section. Underpinning this approach is the capacity to map and monitor the key steps (milestones) that constitute a pathway towards impact. This task requires a high level of cooperation among the main stakeholders in accessing, collecting and interpreting information that supports their ability to make a judgment about the plausibility of the emerging performance story. Ascertaining the attainment of the selected milestones calls attention to the fidelity of the programme as we do not have reason to expect long term impact unless we implement the fellowship programme properly.

The development of a clear pathway implies clear direction. Fellowship programmes that are intended to have impact on the recipient institution should be based on clear analysis of what needs to be developed or strengthened and how training could contribute. Without such direction it is not clear what the fellowship is expected to achieve and to assess its contribution. This information provides a baseline against which progress can be measured and impact determined. If we don't know the situation at the starting point we could not argue that we have added value, nor can we take credit for an improved state of affairs. The initial analysis of training needs provides an assessment of the level of competence before training and enables verification of learning gains. Identification of institutional performance gaps as justification for the fellowship provides a yardstick against which we can measure progress.

Clarity about the recipient system requirements and training needs is essential to guide all aspects of the fellowship programme design, from selection of fellows who are most likely to succeed and contribute, to the design of appropriate training programmes and the preparation of plans for placement and utilisation of fellows on completion of the programme. To be useful, this information must be made available to the host institutions and used to monitor the relevance of the training programme provided. Lack of adequate follow up and support to returning fellows is among the greatest risks to the attainment of the higher level institutional goals, as frequently observed fellows are not given sufficient opportunity to contribute in the areas of expertise they may have acquired. In this way the effectiveness and contribution of the fellowship to the institution is diminished. The contribution analysis may help to identify the disjointed pathway, but beyond a certain timeframe the damage may be irreversible.

To achieve a meaningful progression from analysis and identification of needs to appropriate intervention and subsequent utilisation that leads to sustainable benefits and impact requires a solid partnership between the recipients, the providers and the sponsors of the fellowship programme. As mentioned earlier, one of the key weaknesses of the UN fellowship programme is that most of the important steps are outside the term of reference of the sponsoring agency and thus beyond its control. This weakness needs to be remedied through stronger collaboration and contractual arrangements concerning the execution of the fellowship programme. The active involvement of relevant stakeholder is a cornerstone of the contribution analysis approach. Partnership is required at all steps.

Approaches based on stakeholder participation are built on the principle that stakeholders should be involved in all stages of evaluation, including determining objectives and impacts, identifying and selecting indicators, and participating in data collection and analysis. The stakeholders are essential participants in assessing the contributions towards impact based on the emerging performance story. Their involvement and subsequent ownership of the findings increases the chance that deficiencies would be addressed and opportunities taken up.

## Conclusions

Beyond the high level of commitment and resources required to undertake impact evaluation, it is necessary to recognize three major limitations in evaluating the impact of fellowship programmes:

• Attribution: what would have happened without the intervention?

• Conceptual logic: why is impact expected?

• Fidelity: based on the way the programme was implemented is it justified to expect impact?

Monitoring key milestones from conception to fruition of a fellowship programme mitigates these limitations by assessing the importance and contribution of each step on the logical pathway towards training impact and ascertaining that the fellowship programme has been implemented properly within a logical conceptual framework. Whilst the contribution analysis approach may not be able to provide the casual relations among variables leading to an impact, it could establish a 'plausible association' whereby a 'reasonable person, knowing what has occurred in the programme and that the intended outcomes actually occurred, agrees that the programme contributed to particular outcomes.

The performance story which emerges from this analysis enables 'reasonable' observers to determine the plausibility that particular interventions have led to certain results. The performance story describes the journey from the inception of a fellowship programme to the attainment of its immediate and long term goals. The important events and experiences along the way are identified as milestones that could be monitored in order to ascertain that the programme is leading in the right direction and ultimately that it reached its ultimate goals. The verification that certain milestones have been reached strengthens the confidence that particular contributions helped to attain particular results (attribution) and reduces the uncertainties associated with alternative explanations. In addition, the proposed analysis aims to explore the fidelity of the fellowship programme by ascertaining the fulfillment of necessary steps that justify the expectation of the fellowship producing benefits to the recipient institutions.

The proposed evaluation framework focuses on both qualitative and quantitative evidence concerning the attainment of milestones implied in the extended Kirkpatrick classification of training measures. In addition to the four domains suggested by Kirkpatrick (reaction, learning, behavior, and results) are the pre-training domain of planning, design and implementation, and the long term impact ('mega-impact') following the training programme.

A major advantage of the contribution analysis approach is that it can be based on assessment of any plausible evidence regardless of the design, method or source used to obtain it. Thus, it allows use of information obtained through current monitoring and evaluation approaches and techniques. The use of varied data collection methods provides triangulation of findings.

Accessing existing data sources requires close collaboration with recipient institutions, fellowship authorities and other relevant stakeholders. The task requires a high level of cooperation among the main stakeholders in accessing, collecting and interpreting information that supports their ability to make a judgment about the plausibility of the emerging performance story. Engagement of stakeholders in the elicitation of information and the interpretation of what it means is an essential component of this approach, as we rely on their deeper understanding of contextual issues and conditions which may affect the results. Furthermore, their involvement supports the emergence of ownership, an essential condition for capacity building and lasting improvement.

Effective use of evaluation towards improvement of programmes and determining their merit requires a supportive evaluation culture. Evaluation culture values evidence as a basis for decision making and supports monitoring of outcomes obtained at different stages. Evaluation is viewed as an integral part of planning and managing programmes, from identification of needs, design and implementation of strategies and programmes, to assessing outputs and outcomes towards impacts. At each stage, important decisions are made and information is required. The process is based on partnership and continuing dialogue with key stakeholders about the contributions made by different interventions towards higher level goals.

The recommended approach requires major efforts and investments not undertaken to date. Evaluation does cost but "spending whatever limited funds are made available for a fellowship programme without having any reasonable indication of impact is a waste of much needed resources" [3].

## Senior Fellowship Officers meeting (London, 2008): recommendation

The 17^th ^meeting of the UN Senior Fellowship Officers gave the following verdict on the framework:

"Having considered the various possible evaluation approaches for impact assessment presented during the meeting, by the Task Force experts, led by WHO pursuant to the mandate received during the 16^th ^Senior Fellowship Meeting, and following the deliberations on the resulting findings, the Meeting has found particular merits in the Contribution Analysis approach and therefore adopts this specific modality, with the elaborated milestones pathway as the platform for future implementation and evaluation of Training and Fellowship Capacity Development activities within the UN system" [16].

## Competing interests

The authors declare that they have no competing interests.

## Authors' contributions

AR was primarily responsible for the formulation of the evaluation framework and for drafting the paper. MZ was primarily responsible for the literature review and AG contributed to the formulation of the outline of this review. All three authors contributed to the identification of supportive conditions for implementation of the framework and the formulation of the conclusions. All authors read and approved the final manuscript.

## Supplementary Material

Additional file 1 **Indicators and methods for evaluation of the six stages of fellowship**. Click here for file
