# Supplementary material for: A framework for evaluating the impact of the United Nations fellowship programmes
Source: Hum Resour Health. 2010 Mar 30;8:7. doi: 10.1186/1478-4491-8-7 (PMC2856526; doi:10.1186/1478-4491-8-7)
Supplement: Additional file 1 — Indicators and methods for evaluation of the six stages of fellowship. [file 1478-4491-8-7-S1.DOC]

# Additional file - Indicators and methods for evaluation the six stages of fellowship

The table below provides a summary of sample indicators and methods of data collection that may be used to review the key milestones. Additional indicators may be added or removed to address specific requirements of stakeholders and to accommodate available resources and logistic constraints. The scheme is based on various assumptions. The assumptions may vary but are likely to include the importance of a high level of cooperation among stakeholders and the need to access existing records, reports and other documents.

## Table 1 - Indicators, methods of data collection and assumptions concerning the six stages of the fellowship pathway

| **Stages of evaluation** | **Indicators** | **Methods** | **Assumptions** |
| --- | --- | --- | --- |
| **Program planning, design and implementation**  Most information can be obtained from existing records and documents based on stakeholders cooperation | - Fellowship objectives linked to priority HRD priorities of recipient country/ institution / program - Fellowship based on training needs analysis - Selection criteria and process transparent and efficient - Selection of placement reflects consideration of relevance and efficiency (demonstrated expertise of host institution, other relevant quality measures, location, length of course, and overall cost of fellowship compared to alternative arrangements) - Host institution provides appropriate resources and arrangements to support fellows during study | - Review of records and reports concerning HRD priorities and training needs analysis - Review of selection procedures including: advertising , nomination forms and Minutes of selection committee - Review of host institution experience and expertise in relation to proposed field of study - Review of host institution track record in supporting fellows. - Consultation with stakeholders concerning adequacy of selection and placement | - Close cooperation with recipient and host institutions concerning access to records, documents and personnel. - Willingness of key stakeholders to share information and to make judgment about |
| **Reaction**  Most commonly assessed, relatively easy to address pending cooperation of fellows | - Fellows’ feedback concerning their educational experience and the attainment of their learning goals - % of fellows who have experienced major difficulties and the cause of difficulties encountered (e.g. language proficiency) - % of fellows who would recommend similar training and placement to colleagues - % of fellows who discontinue the training programs (attrition) and the reasons for discontinuation - % of fellows who have completed training within allocated time | - Questionnaires, interviews, focal group discussions with fellows and key support staff in host institutions - Review of records of fellowship completion | - Maintaining contact with fellows following their return - Securing the support of the recipient institution and fellowship authorities to ensure high response rate. - Fellows motivated to respond and to provide detail and thoughtful feedback on their training. - Dealing with response bias associated with self report |
| **Learning**    Relatively easy to assess during training; significantly more difficult the longer the intervals after training | - % of fellows who have attained learning objectives successfully (demonstrated competencies acquired and completed required examinations ) - % of fellows who have not met host institution educational standards and have not been given certificate of attainment | - Testing of knowledge and skills integral to teaching and learning program - Assessment of ability to practice selected skills and apply particular knowledge in different settings | - Assessing gain of competencies due to fellowship based on testing pre-training baseline competencies - Complex learning objectives may be difficult to quantify |
| **Behavior**  Most accurately assessed by observation and interview over time to allow assessment of change | - % of fellows who return home and are employed in relevant positions following training - % of fellows who are able to apply acquired knowledge/skills in work settings (application and transfer) - % of fellows who could demonstrate acquired competencies in work settings. - Perceptions of fellows, supervisors, subordinates and peers concerning enhanced performance and contribution of fellows in the home settings (with tangible examples of such contributions) - % of fellows who have accessed further opportunities for education and professional development. - Increased propensity of fellows to continue to work in home institution/program - % of fellows who have been assigned duties that reflect utilization of their acquired competencies - % of fellows who had their new qualification formally recognized by professional bodies and/or employing authorities for the purpose of remuneration and/or career progression - % of fellows who have demonstrated leadership in their area of work - % of fellows who report improved prospects of career progression - % of fellows who have actively transferred knowledge\skills to others in their institution and beyond (dissemination) | - Review of records and reports - Review of routine performance assessment records and/or initiation of performance assessment focused on expected areas of contribution following the fellowship. - Review of personnel files concerning career progression and other HRH desiderata - 360 degrees survey of fellows and co workers using questionnaires and/or structured interviews and focal group discussion - Observation of job performance using techniques of task/activity analysis - Use of diaries and log books - Records of contributions made by returning fellows to institutional formal and informal continuing education activities | - Difficulties associated with movement of fellows and maintaining contact - Difficulties associated with attrition in longitudinal studies - Securing cooperation of local institution and fellowship authorities in reviewing existing records and reports and initiating supplementary methods of data collection |
| **Result**  Rarely applied due to concerns about attribution, difficulty in assessment and cost involved in systematic case studies | - Change of work practice/procedures/ways of doing things associated with learning - Introduction of new technology - Initiation of a new program or aspects of a program - Evidence of bridging a performance gap in the institution/program related to added capacity - Increasing productivity and coverage - Improved retention and better staff morale - Evidence of contribution to institution key success factors | - Review of records and reports - Consultation with stakeholders - Case studies of institutional success and failure - 360 degrees survey of fellows and co-workers using questionnaires and/or structured interviews and focal group discussions | - Difficulties in attributing contribution of fellowship - Ensuring close cooperation with institution leadership - Quantifying alternative explanations |
| **Mega-impact**  (long Term) | - Addressing national priorities - Improvement of practices at national and global level - Evidence of gains to particular communities as a result of improved services/ programs attributed to contribution of fellows | - Review of records and reports - Consultation with stakeholders - Meta-analysis - Monitoring national indicators | - Difficulties in attributing contribution of fellowship |
